# The paradoxical effects of social class on career adaptability: The role of intolerance of uncertainty

**DOI:** 10.3389/fpsyg.2022.1064603

**Published:** 2022-12-06

**Authors:** Ruimeng Wang, Xinqi Lin, Le Wang

**Affiliations:** ^1^School of Labor and Human Resources, Renmin University of China, Beijing, China; ^2^Department of Physical Education, Shandong University of Traditional Chinese Medicine, Jinan, China

**Keywords:** social class, career adaptability, intolerance of uncertainty, self-esteem, youth career development

## Abstract

**Introduction:**

As a growth background factor, family social class has far-reaching effects on youth career development. However, we have limited understanding of the role and functional mechanisms of social class in career adaptability. Based on the social cognitive theory of social class, we examine the mediating role of intolerance of uncertainty in the relationship between youths’ subjective social class and career adaptability. We also explore the moderating influences of self-esteem.

**Methods:**

Data were collected from a sample consisting of 712 undergraduates (63.2% female) in China.

**Results:**

Results show that subjective social class positively impacts career adaptability *via* prospective anxiety, and negatively impacts career adaptability *via* inhibitory anxiety. The intensity of these indirect relationships is contingent on youths’ self-esteem.

**Discussion:**

Our study illustrates the complex and paradoxical effects of social class on career adaptability and has important theoretical and practical implications. This study expands the theoretical perspective by bringing in the social cognitive theory of social class, provides novel insight into the complex interaction between individuals and the environment in youth career development, and should provide inspiration for the design of career intervention programs.

## Introduction

In recent decades, social class has been shown to have far-reaching impacts on youth career development (Bradley and Corwyn, 2002; Judge and Hurst, 2008; Autin et al., 2017; Kay et al., 2017), with higher social class youths having better career-related achievements (Blustein et al., 2002; Gallo and Matthews, 2003). However, a previous study in the leadership field found being born in a higher social class had a negative effect on the growth of female leaders’ supervisory scope (Li et al., 2011). This reminds us that the story may have two sides. Generally, vocational psychology research focuses on the positive relationship between social class and career-related outcomes, ignoring the disadvantages of being upper class and the advantages of being lower class for youth career development.

Based on the social cognitive theory of social class, this study investigates the paradoxical effect of social class on career adaptability. Career adaptability is defined as “the readiness to cope with the predictable tasks of preparing for and participating in the work role and with the unpredictable adjustments prompted by changes in work and working conditions” (Savickas, 1997, p. 254). We choose it as an indicator of career development primarily because it highlights how individuals deal with unfamiliar situations (Savickas, 1997). This is related to notions based on the above theory that claims social class causes different behaviors in response to unknown circumstances (Johnson et al., 2011; Kraus et al., 2012). Besides, career adaptability is highly valued by employers and researchers. As career instability becomes a normality, it is one of the most important personal qualities for young people seeking to advance in their careers (Guan et al., 2014, 2016). Empirical evidence suggests that career adaptability predicts a variety of positive career-related outcomes, including career construction (Merino-Tejedor et al., 2016) and employment status (Guan et al., 2014). It is worth noting that, because college years are critical to the development of one’s career interests and skills (Malka and Miller, 2007), researchers highlight the key role of this school-to-work transition period for youths’ career adaptability and use the sample of undergraduates to investigate (e.g., Guan et al., 2016; Autin et al., 2017).

This study examines the underlying mechanism between social class and youths’ career adaptability by focusing on the influence of a specific cognitive-person variable, namely intolerance of uncertainty (IU). In line with the social cognitive theory of social class, one’s perceptions of uncertainty are a key point for us to associate social class with career adaptability. Distinct social contexts shape individuals’ cognition of uncertainties who come from upper and lower social class (Johnson et al., 2011; Kraus et al., 2012). In other words, IU may be an important cognitive embodiment of the economic circumstances in which individuals grow up. Meanwhile, one’s perception of uncertainty is an important predictor of behaviors in uncertainty (Arbona et al., 2021). The latter is precisely emphasized in the definition of career adaptability, which is how individuals deal with unknown situations (Savickas, 1997). Thus, IU is likely to be a predictor of one’s career adaptability.

Intolerance of uncertainty is a two-factor structure composed of prospective anxiety (PA) and inhibitory anxiety (IA). The former represents an action-taking tendency in the face of uncertainty, while the latter leads to a tendency to avoid uncertainty (Birrell et al., 2011). This distinction can have the opposite effect on career adaptability. We thereby include the two factors separately to explore the negative and positive pathways underlying the relationship between social class and career adaptability. As stated in the beginning, this study aims to investigate the paradoxical effect of social class.

Social class is a multifaceted construct consisting of two categories: objective and subjective social class. Objective social class refers to a person’s material resources, which are primarily represented by household income, educational attainment, and occupational status (Bradley and Corwyn, 2002); it is already a well-documented predictor in youth career development (e.g., career decision-making; Blustein et al., 2002). Subjective social class refers to “the individual’s perception of his own position in the social hierarchy” (Jackman and Jackman, 1973, p. 569). Recently, researchers have started to emphasize the significant impact of subjective social class on individuals’ career-related outcomes (e.g., work-related learning ability; Thompson and Dahling, 2012). This change is probably because the construct of social class is too complex to be fully represented by a few objective indicators (Autin et al., 2017). Subjective social class is better at reflecting implicit and sensitive information on sociocultural circumstances which cannot be measured by objective indicators (Singh-Manoux et al., 2005; Yan et al., 2021); thus, subjective social class is viewed as a more useful predictor of career-related outcomes (Autin et al., 2017). Based on these findings, the present study examines the unique influence of youths’ subjective social class on their career adaptability by controlling typical objective social class indicators.

Our study adds to the existing literature in the following ways. First, this study goes beyond previous research by exploring the role of a broader contextual factor in predicting career adaptability. Research on the antecedents of career adaptability commonly focused on the impact of intra-individual factors, such as Big Five personality traits and future work self (Guan et al., 2014; Zacher, 2014). Researchers have called for more attention to growth context factors because these can significantly shape one’s career adaptability (Savickas and Porfeli, 2012; Guan et al., 2016). Social class, a typical growth context factor with far-reaching implications, is worth scrutinizing, rather than viewing it as a background variable (Brown et al., 1996; Thompson and Subich, 2006). The current study responds to these calls by highlighting the relationship between social class and career adaptability.

Second, researchers pointed out that due to a tendency to focus on the middle-to-upper class, vocational psychology studies are biased toward the positive consequences of upward mobility (Blau, 1957; Liu et al., 2004; Blustein et al., 2005). The truth may not be as simple as that. The study takes an initial step to realize that a higher social class does not always lead to better career adaptability. This study develops a relatively comprehensive model to deepen our knowledge of the paradoxical impact of social class.

Third, this study expands the theoretical perspective that can explain the relationship between subjective social class and career adaptability by drawing from the social cognitive theory of social class. In limited existing studies, The Psychology of Working Theory is the most common theoretical basis in this regard (Autin et al., 2017; Kim and Allan, 2021). Apart from that, few studies have provided theoretical insights into this relationship. By including the new theoretical perspective, this study can explain the relationship from the cognitive pathway. This should not be neglected because social class and corresponding social contexts shape one’s cognitive disposition, and cognitive factors play a foundational role in one’s adaptive performance (Lent et al., 2002; Li et al., 2011; Kraus et al., 2012). Based on this new perspective, we can also use IU to represent the effect of uncertainty and examine the role of uncertainty directly. This is uncommon but valuable because we are facing a particularly volatile external environment in this era, and uncertainty should not only be underlined in research background.

Fourth, we also go beyond previous studies by identifying the conditions under which subjective social class has more or less influence. Different personal traits cause people to react differently to similar circumstances (Lerner, 1982). Self-esteem is viewed as a type of critical personal resource and has been proven to serve as a protective factor for youth development that buffers the influences of subjective social class (Wang et al., 2016). Moreover, self-esteem may influence the extent to which youths pay attention to uncertainties in the environment because individuals pay different attention to contextual cues among youths with high and low self-esteem (Brockner, 1988). Thus, we submit that self-esteem may moderate the indirect relationship between subjective social class and career adaptability by specifically moderating the relationship between subjective social class and IU.

## Hypotheses and theory

### The mediating role of intolerance of uncertainty between subjective social class and career adaptability

Social cognitive theory of social class provides a cultural perspective on social class. It emphasizes that structural factors, such as educational level or wealth, determine the social context that one lives in and how one is related to others. This implies that people from a similar social context share similar cognitive patterns, which influence a series of thoughts and behaviors (Kraus et al., 2012). According to this theory, upper-and lower-class youths live in different social contexts that influence their development by shaping their cognitive tendencies. The amount and nature of the uncertainty differ between the two social contexts. As such, we propose that subjective social class influences youths’ career adaptability through their response to uncertainties shaped by distinct social contexts.

Intolerance of uncertainty is defined as “the predisposition to react negatively to an uncertain event or situation, independent of its probability of occurrence and of its associated consequences” (Ladouceur et al., 2000, p. 678). IU involves an individual’s negative interpretation of uncertain situations and as such, is viewed as a cognitive disposition (Koerner and Dugas, 2008). Family factors such as parenting behavior can predict IU (Shen et al., 2020). It has also been found to be related to career-related outcomes such as youths’ career decision making (Arbona et al., 2021) and career identity (Garrison et al., 2017).

We propose that subjective social class positively predicts youths’ IU for the following reasons. One of the key differences between upper-and lower-class social contexts lies in uncertainty. The social context of the upper class is characterized by being free and controllable. In contrast, lower-class social contexts are characterized by limited resources and uncertainty (Kraus et al., 2012). These different life circumstances result in upper-and lower-class youths having different perceptions or interpretations of uncertainties. Specifically, for lower-class youths, uncertainty is less likely to induce negative responses because it has a central status in their social contexts. In other words, lower-class youths are more tolerant of uncertainty because it is already a part of their lives, and this possibly promotes their threshold of tolerance of uncertainty. In contrast, when faced with unpredictable events, upper-class youths who believe the world to be stable may be relatively more vulnerable to uncertainty (Lachman and Weaver, 1998), which leads to a stronger intolerance to uncertainty. Therefore, we argue that lower-class youths have advantages in this respect.

Carleton et al. (2007) developed IU as a stable two-factor structure consisting of PA and IA. PA reflects a negative experience of uncertainty as well as a desire for predictability. This implies a proclivity to react actively to reduce uncertainty. IA denotes a mental and behavioral freeze in the face of uncertainty (Berenbaum et al., 2008; Birrell et al., 2011). Although both prospective and IA represent youths’ negative cognition of uncertainty, the differences between them lie in the outcome of cognitive processing.

Individuals with PA may attempt to take actions to reduce the level of uncertainty (Birrell et al., 2011). To increase predictability, people are energized to plan and seek information (Berenbaum et al., 2008). This approach strategy may increase youths’ skills and abilities to cope with novel situations. Moreover, career adaptability itself involves resourceful attitudes and informed decision-making (Savickas, 1997). Thus, we propose that PA is positively correlated with career adaptability. In contrast, youths with high IA are likely to freeze when faced with uncertainty, which results in an inability to apply skills to solve problems and a tendency to avoid uncertainty (Birrell et al., 2011). As such, compared to the approach strategy, this avoidance strategy may lead to a low level of adaptability in career situations.

*H1*: Subjective social class positively impacts career adaptability via PA (*H1a*), and negatively impacts career adaptability via IA (*H1b*).

### The moderating role of self-esteem

Not all individuals are equally influenced by their environment. Personal traits are not a negligible issue when exploring the effect of social class on youths’ career adaptability. Self-esteem is defined as “the overall affective evaluation of one’s own worth, value, or importance” (Blascovich and Tomaka, 1991, p. 115). As stated before, self-esteem is a protective factor of youth development that acts as a buffer against external influences (e.g., poverty). Evidence from cognitive neuroscience suggests that, as a positive psychological resource, self-esteem could buffer the deleterious impact of subjective social class on the gray matter volume that is important for the development of youths’ cognitive capabilities and their future achievement (Wang et al., 2016).

Based on findings from previous studies, we examine the moderating role of self-esteem in the relationship between subjective social class and IU. Youths with high self-esteem have sufficient psychological resources to cope with uncertainties (Hobfoll, 2002), and have a more self-confirmed sense of worth instead of paying much attention to contextual cues (Brockner, 1988), which means they are less likely to be influenced by the number of uncertainties in their life circumstances. For example, despite a lack of uncertainties in their growth environment, upper-class youths with higher self-esteem tend to be less influenced by contextual factors compared to those with lower self-esteem. They have a stronger belief in their own abilities and enough resources to cope with uncertainties. Thus, the positive relationship between subjective social class and IU weakens. In contrast, contextual factors have a stronger influence on individuals with lower self-esteem (Brockner, 1988), so these individuals are more easily influenced by social contexts shaped by social class. At the same time, low self-esteem means that these people have insufficient psychological resources to mobilize to offset these influences. In other words, whether these youths are tolerant of uncertainties is determined more closely by their social class. For example, upper-class youths with lower self-esteem are more likely to be influenced by the lack of uncertainties in their social contexts. They, meanwhile, do not have sufficient confidence and resources to cope with uncertainties.

Taken together, we propose that self-esteem buffers the effect of subjective social class on IU (i.e., PA and IA).

*H2*: Self-esteem moderates the positive relationship between subjective social class and IU, such that both the relationship between subjective social class and PA (*H2a*), and the relationship between subjective social class and IA (*H2b*), are weaker for youths with high self-esteem.

**Figure 1:**
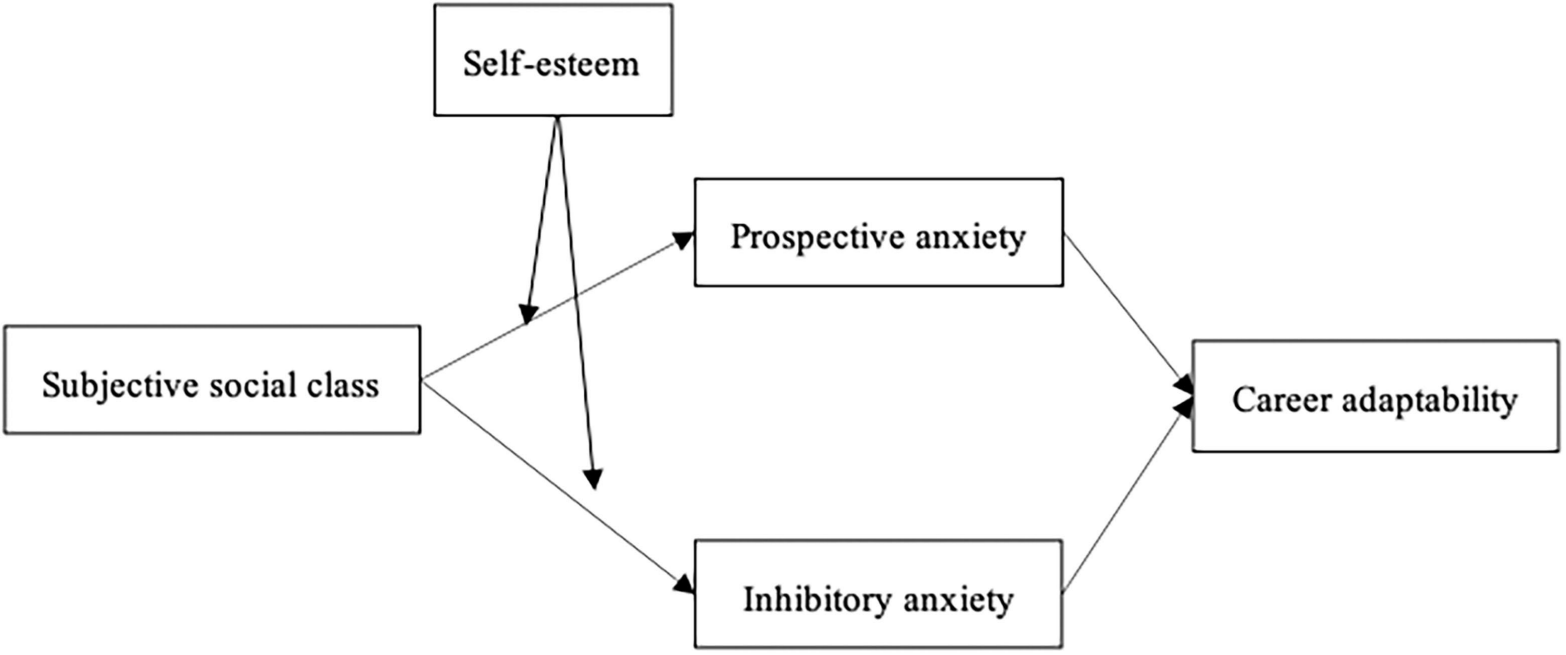
Research model.

The above indicates that the strength of the indirect relationship between subjective social class and career adaptability through IU appears contingent on youths’ self-esteem, which suggests a moderated mediation model (Figure 1).

*H3*: Youths’ self-esteem moderates the strength of the indirect relationship between subjective social class and career adaptability via IU, such that the indirect effect through PA (*H3a*) and IA (*H3b*) are separately reduced for youths with a high level of self-esteem.

## Materials and methods

### Participants and procedures

Data was collected through Wenjuanxing, an online questionnaire distribution platform in China. Online surveys were sent to 892 undergraduates at a large university. The students earned compulsory course credits for participating in this study. At time 1, participants were asked to complete the first part of the survey, which contained a consent form, questions eliciting basic demographic information, and measures of subjective social class and self-esteem in class. At time 2, all participants were invited and asked to complete the second part of the survey, which measured their IU. Career adaptability was measured at Time 3.

After eliminating invalid surveys, 712 (response rate = 79.8%) samples were included in the final analysis. About 63.2% (*n* = 450) of the participants were female and 97.3% (*n* = 693) were aged 18 to 25. 54.2% of the participants were from families with an annual household income of ¥30,000 to ¥100,000 ($4,500–$15,000).

### Measures

#### Subjective social class

We used the MacArthur Scale of Subjective Social Status created by Adler et al. (2000) to measure this variable. A graph with a 10-rung ladder represents where a family stands in the society of China. Each rung corresponds with a number from 1 to 10, which represents ranks of family social class from the lowest to the highest. Participants were asked to place their family on this ladder based on family income, education, and job.

#### Intolerance of uncertainty

This variable was assessed with the 12-item short IU scale developed by Carleton et al. (2007). The sample items of PA included “One should always look ahead so as to avoid surprises.” The sample items of IA included “When I am uncertain, I cannot function very well.” Items were scaled from 1 (not at all) to 5 (very much). A Cronbach’s alpha of.83 was obtained for the subscale of PA, and.90 for the subscale of IA.

#### Self-esteem

This variable was assessed with the 10-item scale developed by Rosenberg (1965). Sample items included “I feel that I’m a person of worth.” Items were scaled from 1 to 4 (1 = strongly agree to 4 = strongly disagree). A Cronbach’s alpha of.93 was obtained for this subscale.

#### Career adaptability

We measured this variable using the 24-item Chinese-version scale developed by Savickas and Porfeli (2012) and translated by Hou et al. (2012). Sample items included “Preparing for the future” and “Exploring my surroundings.” Items were scaled from 1 to 5 (1 = strongly disagree to 5 = strongly agree). The Cronbach’s alpha was 0.98 in this study.

#### Control variables

As stated before, objective social class has influence on individuals’ career development. We controlled for household income and parents’ educational level because they are viewed as the main indicators of objective social class (e.g., Ostrove et al., 2000).

Autin et al. (2017) found that work volition could explain the relationship between social class and CA, so we controlled for this variable as well. The 7-item volition subscale developed by Duffy et al. (2012) were used. Items were scaled from 1 (strongly disagree) to 7 (strongly agree). The Cronbach’s alpha was 0.89.

## Results

### Preliminary analysis

Before testing the hypotheses, we performed a series of confirmatory factor analyses (CFA) using Mplus 8.0 to assess the distinctiveness of the latent variables. We chose the following fit indices to assess the model fit: the relative chi-square (χ2/df) of three or less; Tucker-Lewis Index (TLI) and Comparative Fit Index (CFI) of 0.9 or greater; standardized root mean squared residual (SRMR) and root mean squared error of approximation (RMSEA) of 0.08 or less (Hu and Bentler, 1999).

The four-factor model is composed of self-esteem, PA, IA, and career adaptability. This model offers an adequate fit to the data (χ2/df = 3.24, CFI = 0.99, TLI = 0.98, SRMR = 0.03, RMSEA = 0.06). It is better than the fit of the three-factor model in which PA and IA are combined (χ2/df = 6.06, CFI = 0.96, TLI = 0.95, SRMR = 0.06, RMSEA = 0.08). A two-factor model in which PA, IA, and career adaptability are collapsed into one factor provide a worse fit to the data (χ2/df = 43.18, CFI = 0.67, TLI = 0.56, SRMR = 0.20, RMSEA = 0.24). Finally, a one-factor model where all constructs are combined provides the worst fit to the data (χ2/df = 48.16, CFI = 0.62, TLI = 0.51, SRMR = 0.20, RMSEA = 0.26). These results suggest that the proposed model provides the best model fit, which supports the distinctiveness of the latent variables in this study.

Table 1 presents the descriptive statistics and bivariate correlations in the study. All of the variables are significantly correlated in the expected directions. The results show that youths’ age, gender, and educational level are not correlated with the other variables in the model, so these variables were not included in subsequent analysis.

**Table 1:** Descriptive statistics and correlations.

Variable	*M*	*SD*	1	2	3	4	5	6	7	8	9	10
1. Subjective social class	5.09	1.67										
2. Prospective anxiety	3.68	0.59	0.12**									
3. Inhibitory anxiety	3.23	0.89	0.07*	0.60**								
4. Self-esteem	2.74	0.34	0.09*	−0.17**	−0.44**							
5. Career adaptability	4.13	0.60	0.09*	0.09*	−0.21**	0.39**						
6. Gender	1.63	0.48	0.02	−0.03	−0.01	0.05	0.04					
7. Age	2.97	0.16	0.04	−0.03	−0.05	0.01	0.05	−0.04				
8. EduY	2.98	0.19	0.06	−0.04	−0.06	−0.00	0.01	0.05	−0.02			
9. EduF	2.03	1.11	0.20**	−0.02	−0.09*	0.11**	0.08*	−0.02	−0.07	0.10**		
10. IncomeF	3.30	1.17	0.26**	0.02	−0.05	0.05	0.09*	−0.01	0.06	−0.02	0.24**	
11. Work volition	5.91	0.90	0.11*	−0.04	−0.03	0.11*	0.09*	0.01	0.04	0.04	0.08	0.10*

*N* = 712. EduY, educational level of youth; EduF, the highest educational level of parents; IncomeF, family annual income. *p < 0.05. **p < 0.01.

### Hypotheses testing

To test the above hypotheses, we ran a series of analyses in the PROCESS program with 5,000 bootstrapped samples.

#### Mediation effect

We propose that subjective social class positively impacts career adaptability *via* PA, and negatively impacts career adaptability *via* IA. The results indicate that subjective social class is positively related to PA (*b* = 0.05, 95% CI [0.02, 0.07]) and IA (*b* = 0.06, 95% CI [0.02, 0.10]). That is, the higher social class, the stronger desire for predictability and also the greater likelihood of a behavioral freeze in the face of uncertainties. PA is positively related to career adaptability (*b* = 0.33, 95% CI [0.24, 0.42]), while IA is negatively related to career adaptability (*b* = −0.28, 95% CI [−0.33, −0.22]). The finding supports that due to the stronger willing to take action, youths with higher PA have a greater career adaptability. In turn, due to the higher possibility to freeze in face of uncertainties, higher IA is detrimental to career adaptability. Table 2 shows that the indirect effect of subjective social class on career adaptability through PA (*b* = 0.02, 95% CI [0.01, 0.03]), and separately through IA (*b* = −0.02, 95% CI [−0.03, −0.004]), are significant. Thus, both Hypothesis 1a and 1b are supported.

**Table 2:** Results of mediation effect and moderated mediation effect.

	SSC → PA → CA		SSC → IA → CA	
	*b* (*SE*)	95% CI	*b* (*SE*)	95% CI
Indirect effect	0.02 (0.01)	[0.01, 0.03]	−0.02 (0.01)	[−0.03, −0.00]
				
Moderated mediation effect				
Low self-esteem	0.03 (0.01)	[0.01, 0.04]	−0.03 (0.01)	[−0.05, −0.02]
High self-esteem	0.003 (0.01)	[−0.01, 0.02]	−0.01 (0.01)	[−0.02, 0.01]

SSC, subjective social class; PA, prospective anxiety; IA, inhibitory anxiety; CA, career adaptability; High self-esteem, 1 standard deviation above the mean; Low self-esteem, 1 standard deviation below the mean.

#### Moderation effect

**Figure 2:**
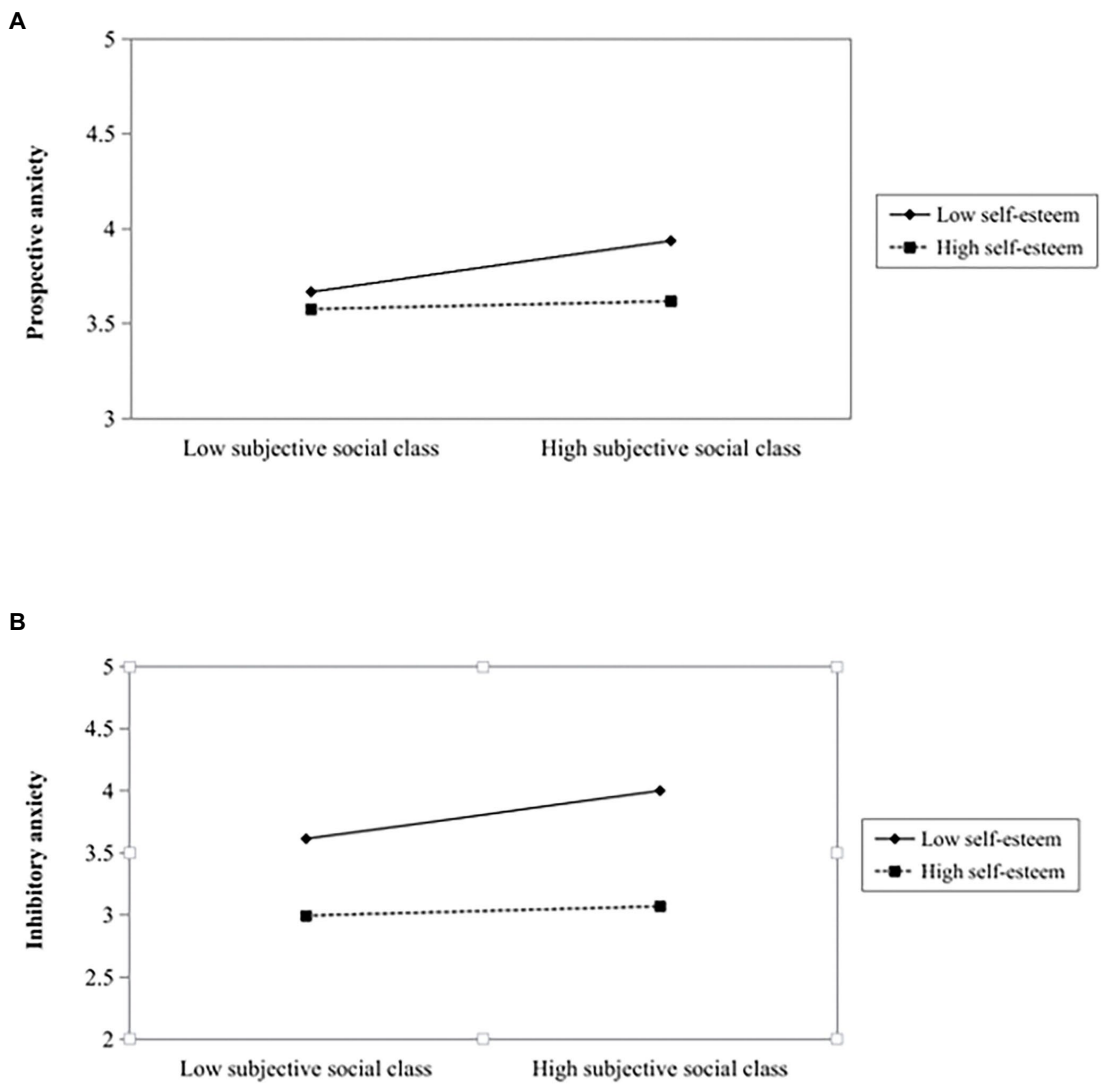
Interaction effect of subjective social class and self-esteem on prospective anxiety **(A)** and inhibitory anxiety **(B)**. High self-esteem, 1 standard deviation above the mean; Low self-esteem, 1 standard deviation below the mean.

Hypothesis 2 proposes the moderating role of self-esteem. As Table 3 shows, the interaction item (subjective social class × self-esteem) has a significantly negative influence on PA (*b* = −0.10, 95% CI [−0.18, −0.03]) and on IA (*b* = −0.15, 95% CI [−0.24, −0.03]). We also performed a simple slopes test. As depicted in Figure 2, the positive relationship between subjective social class and PA (Figure 2A) is strongest at low self-esteem (-1SD, *b* = 0.08, 95% CI [0.05, 0.11]) and weakest at high levels of self-esteem (+1SD, *b* = 0.01, 95% CI [−0.03, 0.05]); and for IA (Figure 2B), the conditional effect is also strongest at low self-esteem (-1SD, *b* = 0.11, 95% CI [0.07, 0.16]) and weakest at high levels of self-esteem (+1SD, *b* = 0.02, 95% CI [−0.04, 0.07]). This suggests that subjective social class has a stronger positive influence on PA or IA in the case of low self-esteem. The higher level of self-esteem, the weaker relationship between subjective social class and PA or IA. Both the two patterns of moderation are consistent with our predictions for the expected buffering effect. Thus, Hypothesis 2a and 2b are supported.

**Table 3:** Self-esteem as a moderator.

Variable	Prospective anxiety		Inhibitory anxiety	
	*b* (*SE*)	95% CI	*b* (*SE*)	95% CI
Predictor variable				
Subjective social class	0.05 (0.01)**	[0.02, 0.07]	0.07 (0.02)**	[0.03, 0.10]
Moderator variable				
Self-esteem	−0.30 (0.06)**	[−0.43, −0.18]	−1.14 (0.09)**	[−1.31, −0.97]
Interaction				
Subjective social class * self-esteem	−0.10 (0.04)**	[−0.18, −0.03]	−0.15 (0.05)**	[−0.24, −0.03]
*R* ^2^	0.06		0.22	

*p < 0.05; **p < 0.01.

#### Moderated mediation effect

The moderated mediation effect posited in Hypothesis 3 is also supported by the results. Table 2 shows that the indirect effects of PA are stronger in the case of low self-esteem (-1SD, *b* = 0.03, 95% CI [0.01, 0.04]) than high self-esteem (+1SD, *b* = 0.34, 95% CI [−0.01, 0.02]). This means that subjective social class has a stronger positive effect on career adaptability *via* PA when youths have lower self-esteem. The moderated mediation effect is also significant (*b* = −0.03, 95% CI [−0.06, −0.01]), supporting Hypothesis 3a. The indirect effects of IA are also stronger in the case of low self-esteem (-1SD, *b* = −0.03, 95% CI [−0.05, 0.02]) than high self-esteem (+1SD, *b* = −0.01, 95% CI [−0.02, 0.01]). Similarly, subjective social class has a stronger negative effect on career adaptability *via* IA when youths have lower self-esteem. Hypothesis 3b is supported as the moderated mediation effect is significant (*b* = 0.04, 95% CI [0.01, 0.07]).

## Discussion

Vocational psychologists have emphasized the importance of environmental factors and their effects on career adaptability (Mayer, 2003; Savickas, 2005). Drawing on the social cognitive theory of social class, this study has established a comprehensive framework that illustrates the complex relationship between social class and youths’ career adaptability by exploring the mediating role of IU and the moderating role of self-esteem.

This study demonstrates the unique contribution of subjective social class by controlling typical objective indicators of social class. Although a limited number of previous studies have explored the relationship between subjective social class and youths’ career adaptability (Autin et al., 2017), the present study makes several significant contributions which extend beyond previous studies.

First, this study makes an important theoretical contribution by bringing the social cognitive theory of social class into the exploration of the relationship between subjective social class and career adaptability. Autin et al. (2017) based their research model primarily on The Psychology of Work Theory, emphasizing the mediating role of work volition. By bringing in a new theoretical perspective, we were able to include a cognitive aspect, namely IU, in the model and examine the cognitive pathway between the two variables.

Career adaptability is viewed as an important quality by employers and scholars precisely because the job market is becoming less certain and stable (Guan et al., 2016). Although uncertainty is frequently mentioned in career adaptability research, it is always regarded as part of the research background. Few studies have directly examined the role of uncertainties. Therefore, the present study contributes to a better understanding of the development of career adaptability by exploring the role that youths’ cognition of uncertainty plays. This also expands theoretical explanations of the relationship between subjective social class and youths’ career adaptability.

Second, the results of the relationship between subjective social class and career adaptability in previous studies are mixed. Autin et al. (2017) found a positive relationship between subjective social class and undergraduates’ career adaptability over time, while no relationship was found between them in other studies among special groups of populations (Douglass et al., 2017). However, the present study differs by developing a comprehensive framework to enable a more precise understanding of the relationship, supporting previous positive findings and also providing a theoretical explanation for unproven negative relations.

The “winner” cannot “take all.” An advantaged social class status does not always lead to a higher level of career adaptability. The results demonstrate that subjective social class affects career adaptability through PA and IA. Subjective social class can lead to PA and serve as a motivating mechanism to promote career adaptability, but it can also lead to IA and have a detrimental effect on career adaptability. Therefore, similarly, the strength in the tolerance of uncertainty that results from lower-class contexts also does not necessarily lead to higher career adaptability. This reflects a complex interaction between individuals and the environment in youth development.

It seems remarkable that subjective social class was found to positively relate to youths’ IU. That is, upper-class youths are more likely to be intolerant of uncertainty than lower-class youths. The results seem to be contrary to previous findings that reported a positive relationship between subjective social class and youth development (Quon and McGrath, 2014). In fact, a qualitative study has demonstrated that youths from economically advantaged families are less likely to make “risky” career choices because they are not sure about the outcome (Lapour and Heppner, 2009). This is consistent with our theoretical hypothesis and also supports our results.

Third, we examined the moderating role of self-esteem, which gave us the opportunity to address how within-person factors and contextual factors work in concert in youths’ career adaptability, which few research studies on career adaptability have focused on. The results show that youths’ self-esteem attenuates the effect of subjective social class on IU and, by this, moderates the indirect relationship between subjective social class and career adaptability. At first glance, it seems that for lower-class youths, high self-esteem makes them more intolerant of uncertainty. Notably, a possible explanation of this result is that high self-esteem is a kind of psychological resource that helps youths cope with uncertainties and, therefore, weakens the promoting effects of lower social class on youths’ tolerance of uncertainty. In other words, the effect of self-esteem offsets the effect of social class on youths’ IU.

Because self-esteem attenuates the relationship between subjective social class with inhibitory and PA separately, the indirect negative effect of subjective social class on career adaptability *via* IA is weakened. Meanwhile, the indirect positive effect of subjective social class on career adaptability *via* PA is also weakened. The moderating role of self-esteem still produces two opposite impacts on career adaptability by moderating both positive and negative mediation pathways. Again, “winners” cannot “take all.”

This study has important practical implications by explaining the paradoxical effect of social class on career adaptability and exploring the mechanism and boundary conditions between the two variables. The results help promote the scientific design of career intervention programs and are meaningful for both youth in the school-to-work transitional stage and practitioners in career development. Policymakers should note that the results demonstrate the important impact of social environmental factors on youth development of career adaptability. Moreover, the results suggest that advantaged social class does not always result in higher career adaptability. This should remind practitioners that only enhancing perceived social class may not be sufficient to help youths to promote their career adaptability. Customized intervention programs for upper-and lower-class youths aimed at their prospective and IA are necessary.

### Limitations and future directions

Despite these valuable contributions, the study has some limitations that could guide directions for future research. First, the relations between these variables are discussed within a cross-sectional framework despite collecting our data from multiple time points. More longitudinal studies are needed to better understand the final developmental result of career adaptability among youths from different social classes. For example, from the perspective of a reserve capacity model, social class advantage leads to more advantageous access to both interpersonal and intrapersonal resources, such as social support and self-esteem (Gallo and Matthews, 2003). These psychosocial resources are found to be helpful to resist stress (Hobfoll, 2002). We infer that this may be one of the explanations for the positive relationship between subjective social class and career adaptability found in Autin et al. (2017) longitudinal study. Thus, future studies could further examine whether and how the reserves, and change in reserves, of psychosocial and material resources of youths from different social classes change the relationship between the variables of this study in the long run.

Second, according to the reserve capacity model, higher social class predicts higher self-esteem. That is, the level of self-esteem among upper-and lower-class groups of youths may originally differ. In this sense, there may be a ceiling effect in the subgroup of upper-class youths when examining the moderating role of self-esteem on youth career development. Meanwhile, the moderating effect might be more salient among lower-class youths. Thus, how the moderating effect differs between the two subgroups of youth could be further explored in future studies.

Third, as already stated, the effect of uncertainty is a focal point as to why we associate social class with career adaptability. We confirmed the mediating role of IU and used it to represent the effect of uncertainty on youths’ cognition. Notably, this just represents a cognitive response to uncertainties. Future studies could further explore if one’s behavioral or motivational responses to uncertainties will mediate subjective social class and career adaptability, deepening understanding of the mechanism between the relationships.

Finally, we asked participants to report family social class because previous research showed that college students are likely to appraise their social class by referring to their parents’ achievements instead of their own (Malka and Miller, 2007). However, the reference object may change with these young people’s growth and they may establish the perceptions of their own social class (Kim and Allan, 2021; Duan et al., 2022). Thus, future studies can also pay attention to this change and adopt various study designs (e.g., situational experimental) to examine what role it plays in youths’ career adaptability. Correspondingly, the measurement of subjective social class should be adjusted to reflect more recent experiences instead of the overall past life experiences measured in this study.

## Conclusion

This study specifically concentrated on the effect of a social structural factor, subjective social class, on youths’ career adaptability, and established a comprehensive framework to explore the paradoxical effect of social class. By bringing in a novel theoretical view, the social cognitive theory of social class, the study examined the role of uncertainty in serving as an underlying mechanism. Subjective social class can lead to PA and serve as a motivating mechanism to promote career adaptability, but it can also lead to IA and have a detrimental effect on career adaptability. Moreover, the intensity of these indirect relations is contingent on youth self-esteem. The study provides novel insight into the complex interaction between individuals and the environment in youth career development, and should provide inspiration for the design of career intervention programs.

## Data availability statement

The raw data supporting the conclusions of this article will be made available by the authors, without undue reservation.

## Ethics statement

Ethical review and approval was not required for the study on human participants in accordance with the local legislation and institutional requirements. The patients/participants provided their written informed consent to participate in this study.

## Author contributions

RW conceived of the study, performed statistical analysis, and completed all parts of the manuscript. XL participated in design and coordination of this study. LW provided part of the financial supports and performed data collection. All authors contributed to the article and approved the submitted version.

## Conflict of interest

The authors declare that the research was conducted in the absence of any commercial or financial relationships that could be construed as a potential conflict of interest.

## Publisher’s note

All claims expressed in this article are solely those of the authors and do not necessarily represent those of their affiliated organizations, or those of the publisher, the editors and the reviewers. Any product that may be evaluated in this article, or claim that may be made by its manufacturer, is not guaranteed or endorsed by the publisher.
